# Accuracy assessment of land cover products in China from 2000 to 2020

**DOI:** 10.1038/s41598-023-39963-0

**Published:** 2023-08-09

**Authors:** Zhiwen Li, Xingyu Chen, Jie Qi, Chong Xu, Jiafu An, Jiandong Chen

**Affiliations:** 1 School of Economics and Management, Southwest Petroleum University, Chengdu, 610500 Sichuan People’s Republic of China; 2 School of Public Administration, Southwestern University of Finance and Economics, Chengdu, 611130 Sichuan People’s Republic of China; 3 Department of Finance and Insurance Faculty of Business, Lingnan University, Tunmen, 999077 Hong Kong People’s Republic of China

**Keywords:** Energy and society, Energy science and technology, Carbon capture and storage, Energy harvesting, Energy infrastructure, Energy storage

## Abstract

The accuracy assessment of land cover data is of significant value to accurately monitor and objectively reproduce spatio-temporal dynamic changes to land surface landscapes. In this study, the interpretation and applicability of CCI, MCD, and CGLS long time-series land cover data products for China were evaluated via consistency analysis and a confusion matrix system using NLUD-C periodic products as reference data. The results showed that CGLS had the highest overall accuracy, Kappa coefficient, and area consistency in the continuous time-series evaluation, followed by MCD, whereas CCI had the worst performance. For the accuracy assessment of subdivided land cover types, the three products could accurately describe the distribution of forest land in China with a high recognition level, but their recognition ability for water body and construction land was poor. Among the other types, CCI could better identify cropland, MCD for grassland, and CGLS for unused land. Based on these evaluation results and characteristics of the data products, we developed suitable selection schemes for users with different requirements.

## Introduction

The accuracy of land cover data is an important basis for global research topics, such as dynamic monitoring of land cover changes, climate and environmental change, regional economic planning, and ecosystem assessment^1,2^. However, owing to the significant area and complexity of terrestrial ecosystems, land cover cannot be directly obtained through field observations during actual use processes; the continuous innovation and development of satellite remote sensing technology provides a new opportunity for the accurate identification of land cover. Compared with traditional field mapping and monitoring, remote sensing monitoring has numerous advantages, such as real-time, objectivity, long time-series, full coverage, traceability, and high precision^3,4^. It has, therefore, become the main technical path for research on global land cover changes^5^. The diverse physical and geographical environment and substantial social and economic activity of China together form a highly heterogeneous and dynamic surface landscape, i.e., higher requirements for land cover mapping, monitoring, and quantitative analysis in this region. Therefore, evaluating the interpretability and accuracy of different Chinese land cover data can aid in objectively recording and reproducing the spatio-temporal dynamic process of land cover changes in China.

Currently, common land cover datasets include the (1) IGBP developed by the international geosphere biosphere program dataset, with a resolution of 1000 m^6^; (2) UMD developed by the University of Maryland, with a resolution of 1000 m^7^; (3) GLC developed by the European Joint Research Center, with a resolution of 1000 m^8^; (4) MODIS land cover dataset (MCD12Q1), with a resolution of 500/1000 m^9^; (5) CCI-LC developed by the European Space Agency, with a resolution of 300 m^10^; (6) CGLS developed by Copernicus land service, with a resolution of 100 m^11^; and (7) NLUD-C developed by the Chinese Academy of Sciences, with a resolution of 30 m^12^. Additionally, some single year or long-term land cover data, as well as many derivatives based on Sentinel and Landsat series satellite data, are also commonly used for research. Although these land cover data provide detailed basic data for examining Earth's surface landscape, significant differences in satellite sensors, monitoring accuracies, classification systems, calibration areas, and applicable environments^13,14^ result in large deviations in the interpretation and accuracy of Chinese land cover.

Presently, there are two techniques to evaluate the accuracy of land cover data. The first method is to establish a confusion matrix that verifies the pixel coincidence degree of land cover data, followed by evaluating the error and omission ratio between the reference data and data for evaluation^10,15^. The sub-pixel confusion matrix (extended on this basis) does not require scale conversion, which largely avoids the uncertainty and error in the processing process^2,16^. The second is consistency analysis, i.e., the area consistency of land cover types of statistical and validation data in corresponding spatial locations^17^. This method reveals the difference and similarity in the spatial distribution of land cover types between the reference data and data for evaluation in a specific area; however, observing specific changes in land cover types is impossible.

Based on the above evaluation methods, many studies have examined the interpretability and applicability of different land cover data in China. (1) Accuracy assessments of China's overall scope, emphasizing verifications of the explanatory power of various land cover data in China at different time points. These studies reveal that GLC2000 products (providing single year data) have strong explanatory power in China with a limited number of remote sensing products^2,18,19^. However, with the increasing availability of high- and medium-resolution satellite images, MCD12Q1 2001/2010 and CCI-LC 2000/2010 also have good explanatory power in China^20^. Generally, most studies lack long-term accuracy assessments, which limits in-depth comparisons and mining of the use value for different data. (2) Accuracy assessment of specific regions in China, i.e., studies that focus on the interpretation of different data for typical regions or individual provinces in China. For example, the verification sample points of typical areas for evaluation are established and selected based on China's physical geography division; the evaluation results for the sample points then reflect the accuracy of different data^21,22^. They can also focus on precision assessments of mixed land cover types or local provinces and cities, such as North China, South China, Northwest China, Wuhan Province, and the black soil region^23–26^. It can reflect the subtle changes of various land cover types in natural location or administrative division, but the research scope is narrow and the conclusion is not universal. (3) Accuracy assessments of China’s sub land types emphasize the applicability of different data to specific land cover types in China, e.g., the thematic precision of cropland, forest, wetland, and other subdivision land types^27–29^. It can compare different products according to specific types, but it is difficult to give a full type of data selection scheme.

Existing research on the interpretability and applicability of land cover data in China has achieved rich results, but the following two deficiencies require further discussion. (1) Presently, accuracy assessments mainly focus on horizontal accuracy comparisons at different time points, focusing on the impact that satellite sources, classification systems, spatial–temporal resolution, and assessment methods of different land cover data have on the accuracy assessment results^13,17^. However, this notably lacks a vertical accuracy trend comparison of long time-series and the impact of the internal characteristics of different data on its accuracy trend, which significantly limits in-depth mining of the applicable features of different data^20^. (2) Previous studies have mostly used global land cover data to evaluate the interpretability of diversified land cover data in specific regions. However, the reference data itself still has a large deviation in the interpretability and accuracy of China's land cover. Simultaneously, without a land classification system suitable for China, carrying out accuracy evaluations according to local conditions is difficult; differentiated evaluation results still require further explanation and discussion.

Based on this review, the research objectives and marginal contributions of this paper had three main points. (1) Combined with Spatial analysis technology, restore the spatial capture comparison view of different land cover data sets from the perspective of “county level”. (2) We not only focus on the horizontal comparison of accuracy between different land cover datasets during the same period or at the same time point, but also pay attention to the comparison of accuracy change trends among different land cover datasets over a long time series, while also taking into account the ‘relative change accuracy comparison’. (3) Reveal the changes in monitoring accuracy of land cover types in China over the past 20 years based on different land cover data, and explain the reasons for the differences in accuracy evaluation of different land cover datasets, taking into account the unique terrain and landforms of China. Propose product usage plans.

## Methodology

### Data sources

#### Data for evaluation

Three land cover data were evaluated in this study. All data were cut uniformly according to China's national boundaries. The details of the three products are as follows: (1) MCD12Q1 data, 500 m resolution from 2001 to 2020; (2) ESACCI data, 300 m resolution from 2000 to 2020; (3) CGLS data, 100 m resolution from 2015 to 2019. The focus of this study was to evaluate the accuracy evolution trend of continuous time series of different products. Thus, the three products selected could not only provide long-term continuous monitoring data but also met the evaluation requirements for resolution differentiation.

#### Reference data

NLUD-C was produced by the Chinese Academy of Sciences (http://www.resdc.cn). When updating data from each year, the remote sensing images, manual visual interpretation, field investigation and a large quantity of auxiliary information and other measurement means were used comprehensively. This product pays more attention to the thematic accuracy and positioning accuracy of land cover change dynamics. It is the most accurate remote sensing product known for explaining China's land cover types^30^.

#### Data preprocessing

The unified classification scheme is an important prerequisite for evaluating the interpretability of different land cover data. Based on NLUD-C classification standard, as well as by referring to the classification methods of Latifovic and Olthof^16^ and Herold et al.^13^. We unified the classification system: cropland, forest, grassland, water bodies, construction land, and unused land (Table 1). The transformation of the classification system mainly followed two principles: (1) the principle of approximate area, i.e., the area of land cover types with different data after statistical classification and (2) view consistency principle, i.e., observing the spatial position relationship between different land cover types and various types of reference data after classification. Taking three land cover data for evaluation as the benchmark, the nearest neighbor matching method was used to capture and resample the reference data to unify the pixel size and prevent patch drift from impacting the evaluation results.

**Table 1:** Transformation of the classification system for the three types of land cover datasets.

NLUD-C	CCI	MCD	CGLS
1. Croplands	Cropland, rainfed, Cropland, irrigated or post-flooding	Croplands, Cropland/Natural Vegetation Mosaics	Cultivated and managed vegetation/agriculture
2. Forest	Mosaic cropland/natural vegetation, Tree cover, broad leaved/needle leaved, evergreen/deciduous, closed to open (> 15%), Tree cover, mixed leaf type, Mosaic tree and shrub (> 50%)/herbaceous cover (< 50%), Shrubland	Evergreen Needleleaf/Broadleaf Forests, Deciduous Needleleaf/Broadleaf Forests, Mixed Forests, Closed/Open Shrublands, Woody Savannas, Savannas	Shrubs, Closed forest, evergreen needle/broad leaf, Closed forest, deciduous needle/broad leaf, Closed forest, mixed/unknown, Open forest, evergreen needle/broad leaf, Open forest, deciduous needle/broad leaf, Open forest, mixed/unknown
3. Grassland	Mosaic herbaceous cover (> 50%)/tree and shrub (< 50%), Grassland	Grasslands	Herbaceous vegetation
4. Water Bodies	Tree cover, flooded, fresh or brackish water/saline water, Shrub or herbaceous cover, flooded, fresh/saline/brackish water, Water bodies, Permanent snow and ice	Permanent Snow and Ice, Water Bodies	Snow and Ice, Permanent water bodies, Open sea
5. Urban and Built-up	Urban areas	Urban and Built-up Lands	Urban and Built-up
6. Unused land	Lichens and mosses, Sparse vegetation (< 15%), Bare areas	Permanent Wetlands, Barren	No input data available, Bare/sparse vegetation, Herbaceous wetland, Moss and lichen

### Analytical methods

#### Consistency analysis

The consistency test is often used to calculate the consistency of different research objects. This study used the area proportion of the same land cover type in the corresponding spatial position of different data, which is used to verify the similarity between the area of the land cover type in the data for evaluation and the area of the corresponding type in the reference data, as expressed by the consistency coefficient^18,31,32^:

1 $$ A_{0} = \frac{{\sum\limits_{i = 1}^{n} {XY_{ii} } }}{M} \times 100\% , $$

where $X_{i}$ represents the area of the class $i$ land type in data product $X$, $Y_{i}$ represents the area of the class $i$ land type in data product $Y$, $XY_{ii}$ represents the same area of the class $i$ land type in data $X$ and $Y$, $n$ is the number of classifications, and $M$ is the total area of the study area. Equation (1) can also be used to calculate the individual area consistency, $A_{i}$, and the overall area consistency of the class $i$ land type.

#### Accuracy evaluation

The confusion matrix, also known as an error matrix, is an important application method for land cover accuracy evaluation. It can intuitively reflect the classification relationship between the data for evaluation and the reference data. Its specific evaluation indicators include the Kappa coefficient, overall accuracy, producer accuracy, and user accuracy, among others. These accuracy indicators can reflect the accuracy of the land cover data product classification from different dimensions^33^. The Kappa coefficient is an important reference index for accuracy evaluations:

2 $$ K_{hat} = \frac{{N\sum\limits_{i = 1}^{n} {T_{ii} } - \sum\limits_{i = 1}^{n} {\left( {T_{i + } T_{ + i} } \right)} }}{{N^{2} - \sum\limits_{i = 1}^{n} {\left( {T_{i + } T_{ + i} } \right)} }}, $$

where $N$ is the total number of verification points or samples for the accuracy evaluation in the confusion matrix, $n$ is the type number of land cover data in the confusion matrix, $T_{ii}$ is the number of land types correctly classified (the sum is the sum of the diagonals in the confusion matrix), $T_{i + }$ represents the sum of the category in the classified data, and $T_{ + i}$ represents the sum of category $i$ in the measured or reference data.

## Results

### Visual comparison

**Figure 1:**
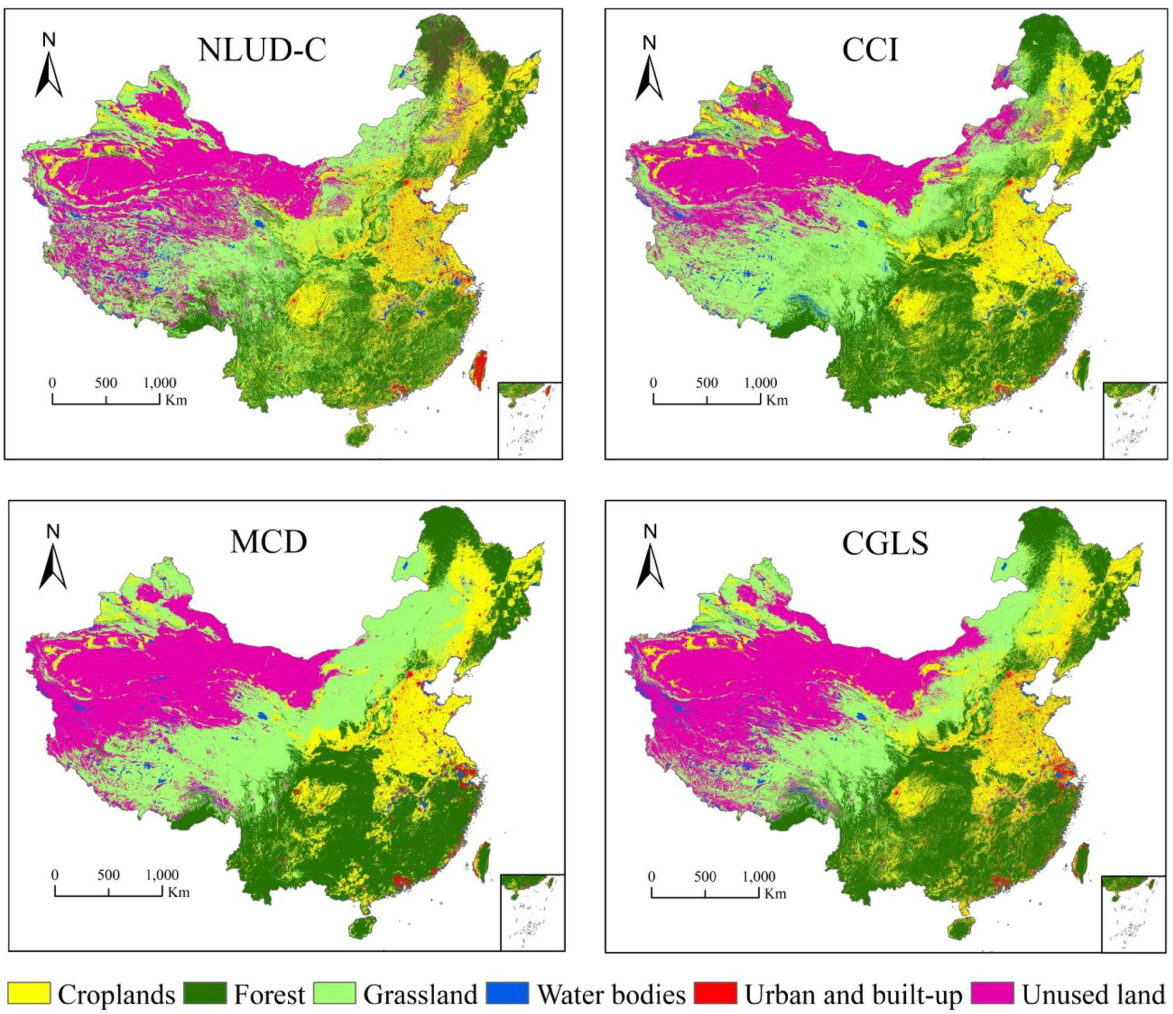
Visual comparison of the four types of land cover datasets in 2015.

Based on the land cover classification system for NLUD-C products, we converted the CCI, MCD, and CGLS land cover data into the NLUD-C classification system via GIS reclassification technology. Considering that the period of the data for evaluation in this paper was inconsistent, as well as that the common year for all of the data was 2015, we provided classification and transformation views of the four land cover data types in 2015 to preliminarily show the spatial location comparison of different land cover data, as exhibited in Fig. 1. Different land cover data could approximately reveal and describe the spatial characteristics and distribution of land cover in China, such as forest in the south, cropland in the middle east, and unused land in the northwest. There were still many visual differences between the data for evaluation and the reference data, especially in the distribution description for the mixed areas of land cover types, such as northern China represented by the Inner Mongolia Plateau, mountainous areas in southwestern China represented by Tibet, and the forest grass mixed zone in the inland areas of the middle east.

### Consistency analysis

Table 2 lists the overall consistency results for the three land cover data for evaluation during the study period. The overall consistency level of the three land cover data fluctuates between 65 and 70%, which is highly consistent with the reference data. Specifically, in the comparison for 2005, 2010, and 2020, the overall consistency of the MCD was slightly higher than that of the CCI. The CGLS had the highest overall consistency in the horizontal comparison in 2015 and the strongest description of China's land cover distribution. From the time series, the overall consistent level of CCI decreased slightly, while MCD increased slightly, but both changes fluctuated stably.

**Table 2:** Changes in the overall area consistency values from 2000 to 2020.

Type	2000	2005		2010		2015			2020	
	CCI (%)	CCI (%)	MCD (%)	CCI (%)	MCD (%)	CCI (%)	MCD (%)	CGLS (%)	CCI (%)	MCD (%)
Croplands	70.61	70.82%	58.09	71.15	57.90	70.70	58.85	66.78	70.04	59.35
Forest	80.81	81.00	80.22	79.90	79.18	79.88	79.81	82.32	80.37	80.10
Grassland	56.73	57.14	61.58	57.04	63.95	57.51	63.92	56.25	56.51	63.59
Water	51.83	51.84	34.82	49.95	34.08	50.23	34.34	46.33	50.10	34.90
Urban	17.57	24.94	28.36	31.34	28.10	33.76	28.10	46.51	39.99	31.53
Unused	66.59	66.30	78.18	62.72	75.73	62.14	75.31	79.80	63.22	75.63

Table 2 shows the comparison of consistency results of subdivided land cover types. Compared with the reference data, the consistency level of forest in the three products is maintained at about 80%, and the similarity is high. The consistency level of croplands in CCI is the best, that of grassland and water is the best in MCD, and that of unused land in CGLS is slightly better than MCD and much higher than CCI. Although the consistency of construction land in CGLS is better than CCI and MCD, the similarity of the three products is low. Among them, the consistency difference of water bodies is the most prominent, reaching the maximum difference of 17.20% in 2005. CGLS was added to the evaluation sequence in 2015, and the consistency difference of construction land was the largest, reaching 18.41%, followed by unused land and water bodies.

From a vertical trend perspective, the consistency of cropland, forest, grassland and water bodies among the three products was relatively stable, while the construction land and unused land had notable changes. The consistency level of construction land in CCI increased from 17.57% in 2000 to 39.99% in 2020, with a large and rapid increase, and the consistency difference range between CCI and MCD is expanding. However, compared with other land cover types, the consistency level of construction land is still low. The consistency level of unused land shows a slow downward trend, but the decline of CCI is more notable than that of MCD.

### Accuracy evaluation

**Figure 2:**
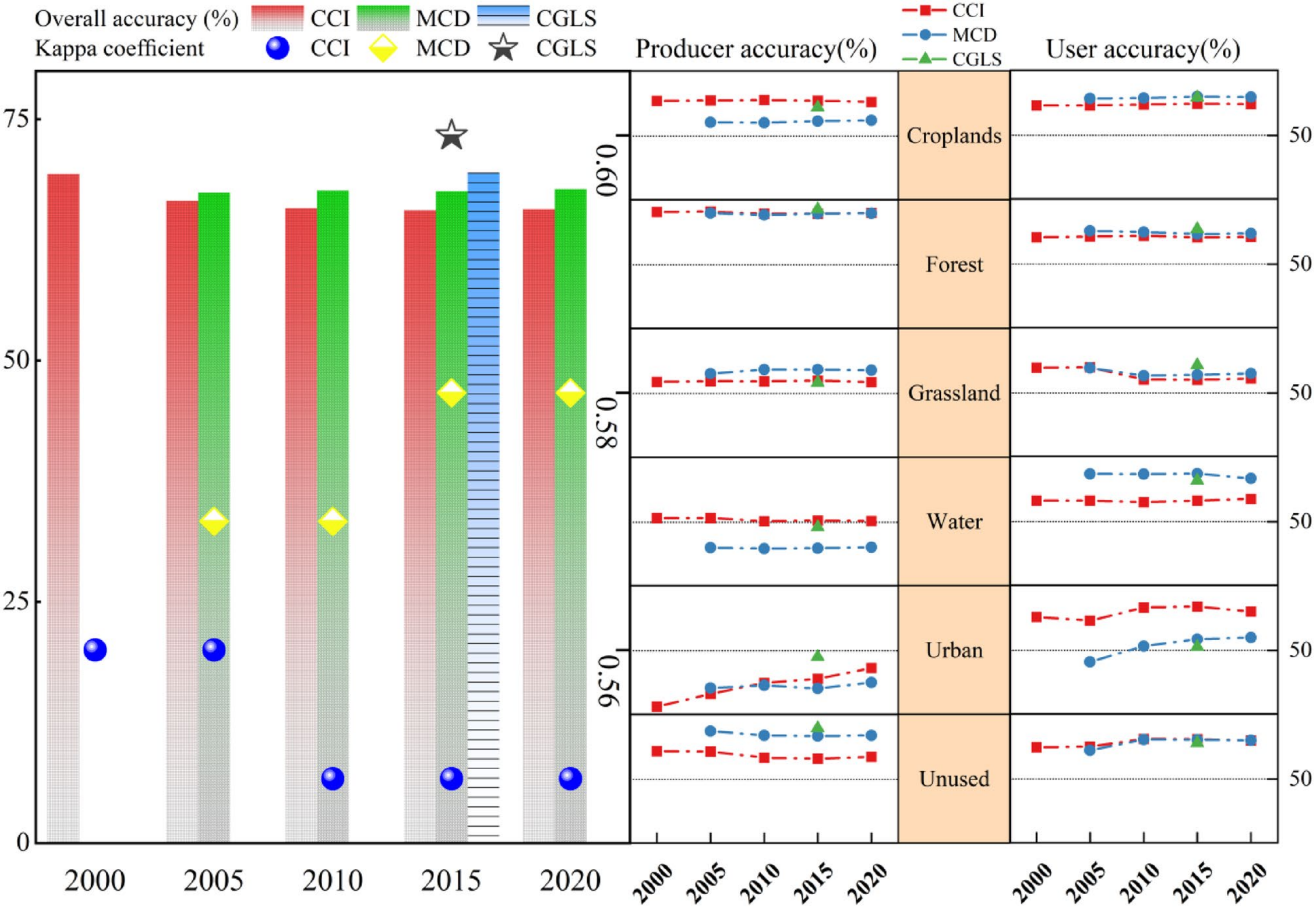
Combination of overall accuracy (from 0 to 75%, see the left half of the figure), Kappa coefficient (from 0 to 1, see the left half of the figure), producer and user accuracy (from 0 to 100%, see the right half of the figure) for land cover datasets.

Based on the confusion matrix, Fig. 2 shows the user accuracy, producer accuracy, overall accuracy and Kappa coefficient of the three products. CGLS had the highest overall accuracy and kappa coefficient, which were 69.45% and 0.60 respectively, CCI had the lowest overall accuracy and kappa coefficient, which were 65.56% and 0.55 respectively, and MCD was between these two. This is mutually confirmed with the conclusion of the above area consistency.

In the comparison of the producer precision (Fig. 2), the three products depicted the actual situation for forest cover in China with a high recognition level of approximately 80%. Among them, the CCI could better map cropland; the production mapping level of water bodies was higher than the MCD and CGLS. The MCD could better map unused land; the production mapping level of grassland was higher than the CCI. Mapping accuracies of 82.36 and 79.87% for the CGLS products in 2015 were closer to the actual situation for forest and unused land cover in China. Overall, the three products had a very weak recognition ability for construction land; the MCD and CGLS had a weak recognition ability for water bodies. Based on the confusion matrix, the identification confusion between water bodies, construction land, and other land was the reason for the low accuracy of the two producers. From a vertical trend perspective, the identification ability of the CCI for construction land has significantly improved since 2000, from 17.27 to 40%, but it is still at a low level. The precision of all land cover types in other products maintained a stable fluctuation range.

In the comparison of the user accuracy, construction land and unused land were highly available in the CCI, cropland and water were highly available in MCD, and forest and grassland were the highest in the CGLS. From a vertical trend perspective, the user accuracy of construction land in the CCI has increased from 69.48 to 72.65% since 2000; the difference between the user and producer accuracy has decreased, indicating that the credibility of construction land in the CCI has gradually improved. Only the user accuracy of grassland shows a downward trend in CCI, and the user and producer accuracy remained at a similar low value, which indicated that the probability of a misclassification and missed scores was similar, and the monitoring level must be improved. Since 2005, the user accuracy of construction land and unused land in the MCD has increased significantly. Construction land has increased from 43.37 to 57.55%, while unused land has increased from 66.83 to 72.66%.

### Relative accuracy evaluation

#### Reference data preprocessing

**Figure 3:**
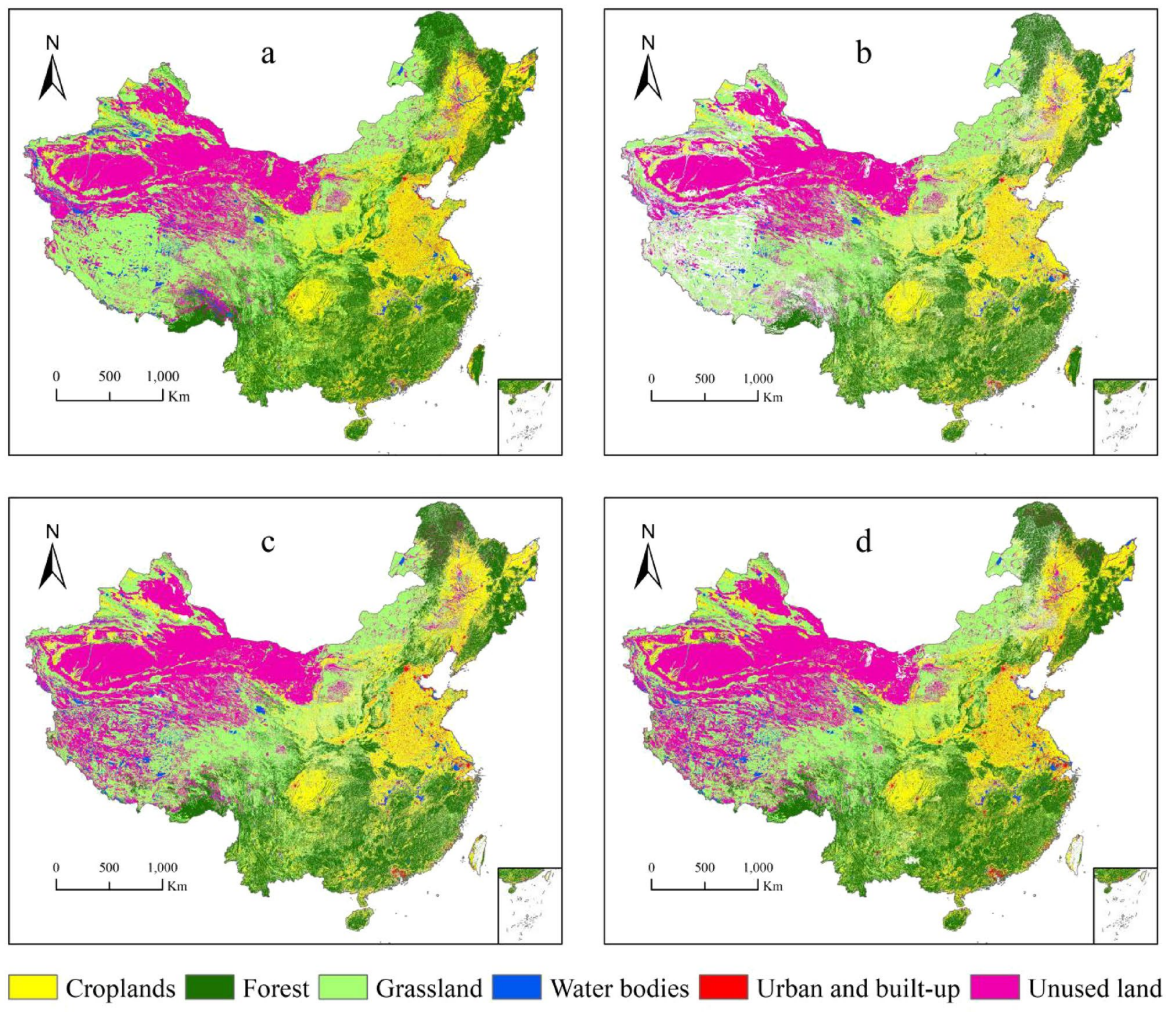
Relative accuracy reference data view. (**a**) Represents the evaluation reference data from 2001 to 2004. (**b**) Represents the evaluation reference data from 2006 to 2009. (**c**) Represents the evaluation reference data from 2011 to 2014. (**d**) Represents the evaluation reference data from 2016 to 2019.

As the NLUD-C product only provides a dataset every 5 years, as well as considering the realistic background of small land cover changes in adjacent years, we assumed that the land cover type would not change significantly within 5 years, taking the unchanged areas and types as a reference for evaluating the relative change in accuracy of other land cover data in the same period. We then carried out consistency analysis and established a new confusion matrix for evaluation. Figure 3 shows the regional view of China's land cover that did not change during the four periods of the NLUD-C product, i.e., the reference data for the relative accuracy evaluation. The blank area represents the area where the land cover type has changed.

#### Relative consistency analysis

**Figure 4:**
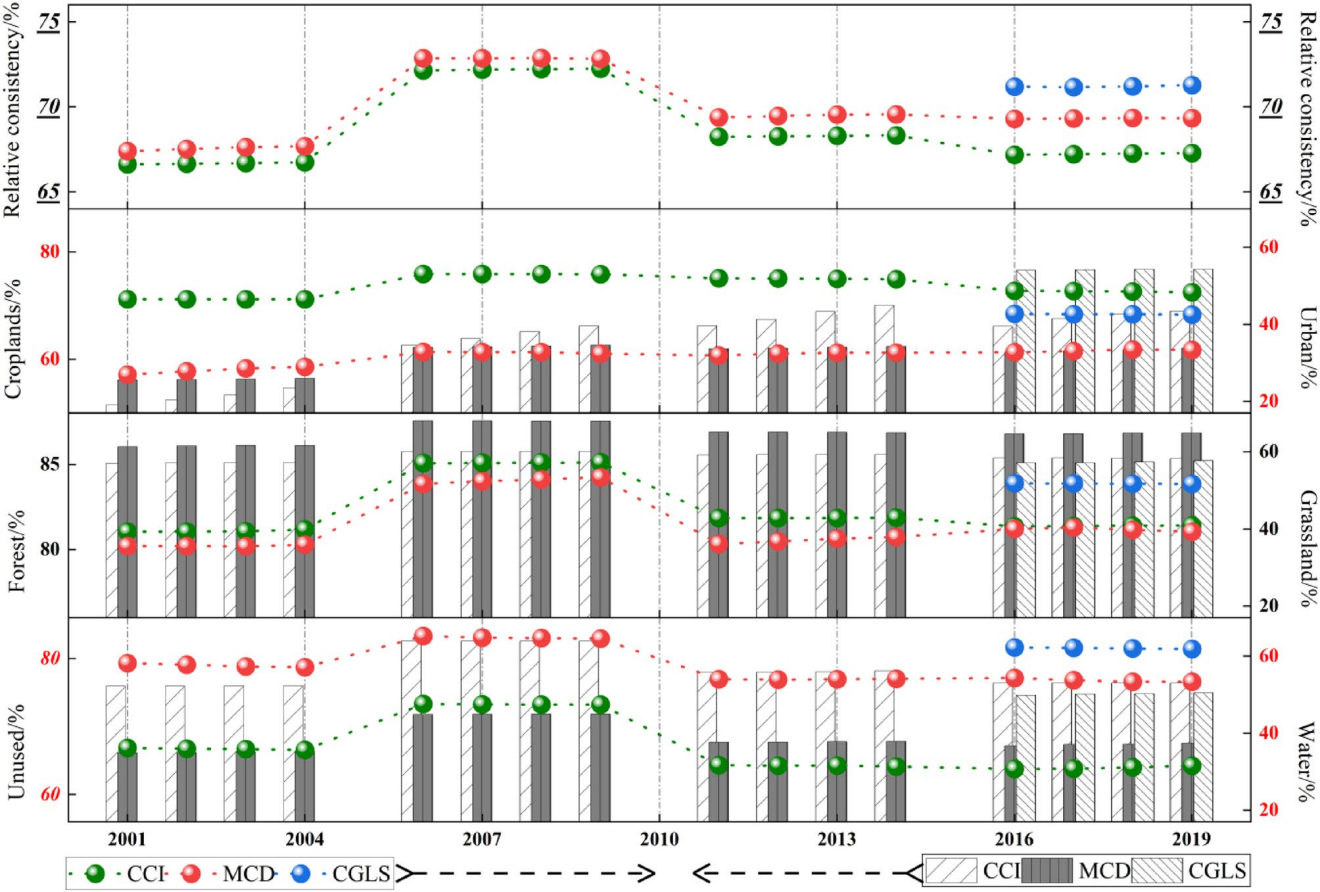
Consistency of the relative area from 2001 to 2019. The “relative consistency” at the top of the figure represents “overall area consistency from a relative perspective”. The other parts in the figure represent the relative area consistency of land cover classification, and the arrow in the legend below points towards the reference direction of the vertical coordinate. For example, the consistency interval of unused land is 60–80 or above.

In the relative consistency analysis (Fig. 4), the overall area consistency of the MCD in each period was always higher than that of the CCI. The relative consistency level of 2001–2004, 2011–2014, and 2016–2019 fluctuated between 65 and 70%. In the horizontal comparison for 2015–2019, the relative consistency level of the CGLS was the highest, agreeing with the consistency analysis results for the time-sharing points above. We note that the relative consistency between the CCI and MCD from 2006 to 2009 was significantly higher than that in other periods.

The results for the relative consistency of different land cover types in different products were basically the same as the previous consistency analysis conclusions, but the trend difference and degree of relative consistency for the various types across the periods were more prominent. As shown in Fig. 4, the relative area consistency of forest was still the highest, while the relative consistency for construction land in all periods for the CCI showed a notable growth trend, with the fastest growth rate from 2011 to 2014. The range of the relative consistency difference between the CCI and MCD was the largest from 2001 to 2014 and highest at 19.03% in 2006. With the addition of the CGLS to the research sequence, the consistency difference degree of construction land became first, reaching 21.23% in 2016, followed by unused land and water, with the highest reaching 17.84 and 16.29%, respectively. This indicates that the nature of construction land, unused land, and water changed significantly during this period; the detection level of the CGLS for construction land and unused land was significantly higher than that of the CCI and MCD.

#### Relative accuracy analysis

**Figure 5:**
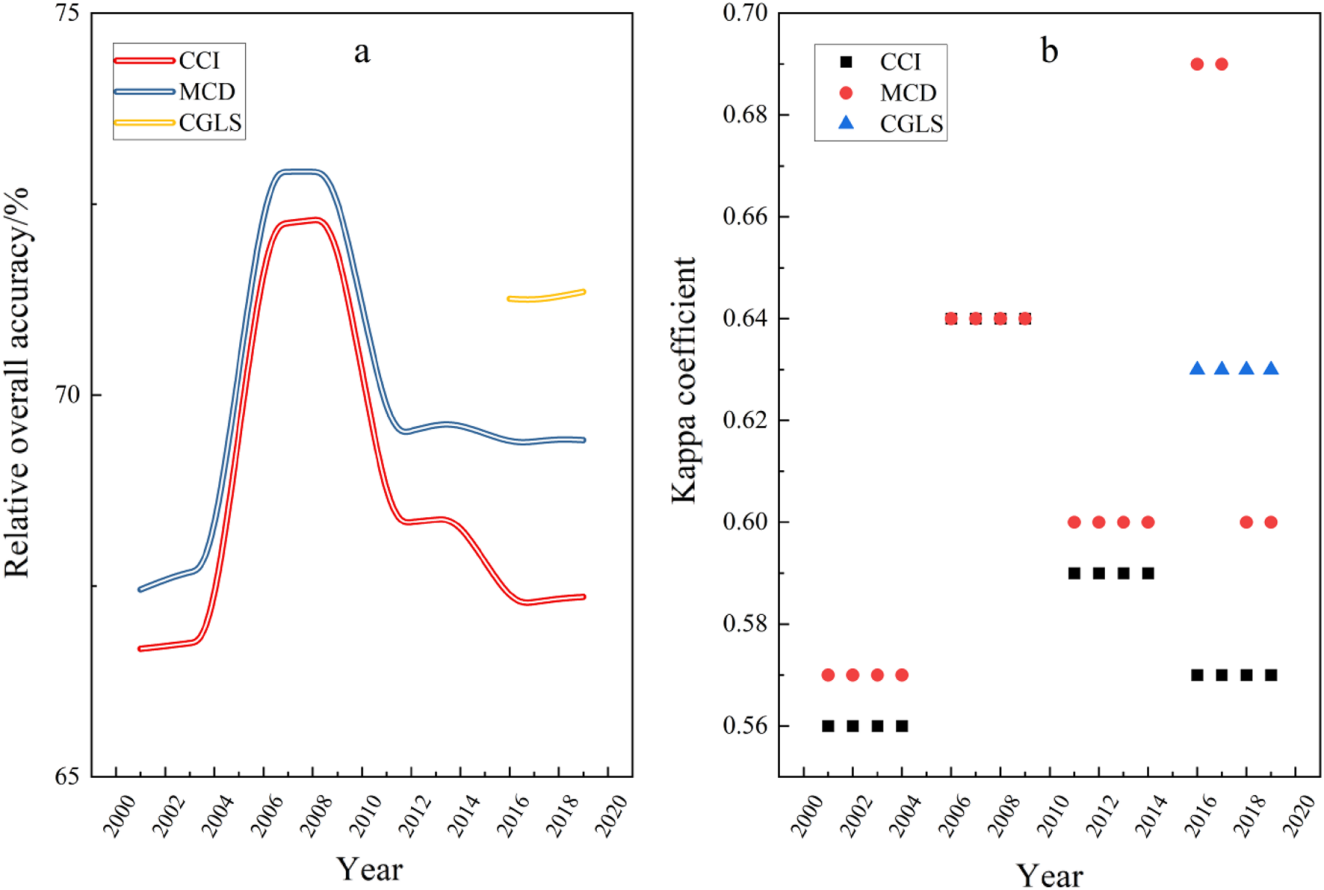
Relative overall accuracy (**a**) and Kappa coefficient (**b**).

Figure 5 showed the results of the confusion matrix in each period of study. The overall accuracy of CGLS were better than those of MCD and CCI, the relative accuracy level of CCI was the lowest, and the accuracy change trend of the three products remained stable. The overall accuracy and kappa coefficient from 2006 to 2009 were significantly higher than those in other periods, which indicates that there are still inherent misclassification and missing points in the monitoring of unchanged areas in China by land cover data, and the monitoring capacity and level have not been significantly improved. We noted that the Kappa coefficient of water bodies in 2016–2019 showed a fault type difference in MCD. Combined with the Kappa coefficient calculation formula and the following user accuracy observation, the fault phenomenon originated from the continuously increasing number of misclassified samples for water bodies and construction land in the MCD.

In the trend comparison of the producer precision (Table 3), the producer precision of cropland, forest, grassland, water bodies, and unused land in different products remained stable. Only the production identification level of construction land in the CCI showed a notable growth trend; construction land in the MCD also increased slightly. At the same time, the producer precision of construction land for other products was relatively stable during this period. Based on Table 3, the precision of most producers of various land cover types during 2006–2009 was significantly higher than that in other periods, which not only showed that China has a large land area that did not change from 2005 to 2010, but also showed the importance of the detection precision of individual land cover types to the overall evaluation precision of products.

**Table 3:** Relative accuracy of different types of producers and users.

	CCI		MCD		CGLS		CCI		MCD		CGLS	
	PA	UA	PA	UA	PA	UA	PA	UA	PA	UA	PA	UA
	Croplands						Forest					
2001–2004	71.16%	67.96%	57.95%	71.84%	–	–	81.13%	66.28%	80.26%	69.56%	–	–
2006–2009	75.86%	74.03%	61.28%	76.99%	–	–	85.14%	72.27%	84.10%	73.14%	–	–
2011–2014	74.99%	72.49%	61.06%	75.15%	–	–	81.89%	70.12%	80.58%	70.97%	–	–
2016–2019	72.64%	70.49%	61.59%	74.53%	68.39%	74.65%	81.42%	68.33%	81.21%	70.28%	83.91%	73.31%

	Grassland						Water bodies					
2001–2004	57.23%	65.09%	61.57%	65.00%	–	–	52.92%	62.82%	35.50%	80.58%	–	–
2006–2009	60.16%	67.31%	68.08%	67.57%	–	–	64.90%	71.10%	45.63%	85.22%	–	–
2011–2014	59.30%	59.13%	65.15%	61.88%	–	–	56.71%	64.43%	38.23%	79.70%	–	–
2016–2019	58.39%	59.04%	64.83%	62.46%	57.46%	68.41%	53.81%	64.39%	37.61%	77.63%	50.90%	75.30%

	Urban and built-up						Unused land					
2001–2004	21.12%	68.72%	25.76%	39.96%	–	–	66.74%	68.87%	79.03%	66.77%	–	–
2006–2009	37.16%	78.14%	34.31%	49.71%	–	–	73.31%	76.46%	83.12%	77.06%	–	–
2011–2014	42.33%	80.73%	33.95%	56.18%	–	–	64.27%	74.53%	77.01%	73.95%	–	–
2016–2019	41.80%	76.88%	33.35%	58.71%	54.19%	54.04%	64.00%	73.79%	76.83%	73.57%	81.58%	71.00%

Where PA represents producer precision, UA represents user precision. Due to space limitations, the values in the table represent the average level of each time period.

In the trend comparison of the user accuracy in different periods (Table 3), the availability of construction land and unused land was high in the CCI; the availability of cropland, forest, grassland, and water bodies was high in the CGLS, followed by elevated performance in the MCD. From the longitudinal trend of each study period, the user accuracy of grassland in CCI and water in MCD decreased slightly, that of unused land in the CCI and MCD increased slightly, and the user accuracy of construction land in the MCD showed a notable growth trend. In the three types of land cover data, the producer precision of forest was significantly higher than the user precision; the user precision of water bodies and construction land in the CCI and MCD was significantly higher than the producer precision. This indicates that forest had the lowest missing probability and highest recognition accuracy; however, water bodies and construction land had the highest misclassification probability and lowest recognition accuracy.

## Discussion

### Other factors that affect how accuracy is assessed

First, during data processing, resampling caused the inherent pattern deviation in the land cover view, especially in the pixel mixing area. This yielded inevitable errors in the accuracy evaluation results. Subpixel evaluation approaches can reduce mistakes, according to prior studies, but they are still limited by the evaluation scale and sample validation interval^2,16^. Second, in addition to monitoring scale differences in the product data sources, most of the evaluation errors and errors in the three land cover data mainly originated from inconsistencies in the classification schemes and evaluation methods, which were less affected by resolution differences^34^. This is especially evident in the hazy definition of land cover subdivision kinds in various products, but it is challenging for production institutions to build a consistent definition system due to the complexity of coordinating many product usage objects and application sectors^35^.

### Differences in evaluation between CCI and MCD products

In this article, MCD products have high overall consistency and producer accuracy^18^, while CCI products have great overall accuracy in earlier horizontal comparison studies. Although the CCI product using the LCCS classification system is better suited for the complexity of the world's land cover^23,36,37^, it is ineffective for China, which is primarily located in mid-latitude regions because it overemphasizes the “advantages” of land classification attributes. Although some studies set the evaluation area as China^20,38^, the unified processing for the classification system of data for evaluation did not follow the NLUD-C products, which fundamentally affected the evaluation process and calculation results. Comparatively, CGLS products had a high resolution and subdivision of land attributes.

### Identification differences in subdivided land classification

In the assessment of land cover types, cropland, grassland, and forest were sensitive to spectral reflection^1,39^, i.e., they were easily monitored, classified, and retrieved by remote sensing images. Therefore, these three types of land remained in a relatively stable precision assessment range; however, the construction land, unused land and water bodies with abnormal differences require detail discussions. The actual spatial distribution of land cover in China does not have strict boundaries, especially in the construction land that includes both urban and rural land. Therefore, low resolution products are difficult to effectively identify rural construction land, as it is easily confused with farmland, grasslands, and other land. Moreover, the extraction and identification methods for construction land differentiation by different products mainly affect the assessment results, such as CCI identifying construction land based on constantly updated MODIS data^10^ or MCD monitoring of construction land changes based on central verification points^9^. (2) The resolution is a direct factor affecting the monitoring and identification of the spatial position and area of a water bodies. When the resolution was increased from 500 to 100 m, the accuracy level of the CGLS water bodies was significantly higher than that of the MCD. However, the water bodies monitoring level for the 300 m resolution CCI product in the classification accuracy assessment was significantly higher than that of the CGLS because the ENVISAT satellite adopts non-optical image recognition for water bodies. (3) The heterogeneity of classification systems is the objective reason for significant differences in the accuracy evaluation of unused land. Due to the fuzzy intersection between different product classification systems in terms of type, land attributes, regional boundaries, and unused land classification, the number of misclassified and missed samples for unused land is higher than that of other land cover types.

### Limitations and prospects

The limitation of this paper is that although consistency and precision calculation are good evaluation indicators, the accuracy evaluation level of this paper is only limited to the first level categories such as cultivated land, forest land, etc., and it fails to completely correspond to the subdivision of land types, such as evergreen broad-leaved forest, paddy field, savanna, etc. In future research, it is worth discussing in depth the accuracy monitoring comparison of China's segmented land types. Repeatedly verifying and matching the differentiated classification indicators of different land cover datasets before formal comparison is an important prerequisite for increasing the scientific and accurate accuracy of accuracy comparison. At the same time, predicting land cover changes based on the characteristics of different datasets and applying them to other fields is particularly important, such as carbon emission assessment or ecological indicator assessment.

## Conclusions

Based on NLUD-C products, we used consistency analysis and a confusion matrix to evaluate the accuracy level of three types of long-term time-series land cover data. Specifically, we first took the time period of nlud-c as the node to carry out the horizontal accuracy comparison and evaluation of different data. Secondly, we further carried out a comparative analysis of the relative accuracy trend for the three types of data in different periods. The main conclusions are as follows: (1) Overall accuracy, Kappa coefficient, and consistency level of different products. The CGLS data had the highest overall accuracy, Kappa coefficient, and area consistency in the accuracy evaluation of the time points and periods. The overall accuracy of the CCI data and Kappa were the lowest, and the area consistency level was poor. The MCD data was in between. (2) Consistency level, precision status, and trends in land cover classification. CCI is better at identifying and mapping cropland and water, while MCD can better identify and map grassland and unused land. Compared with the CCI and MCD, CGLS showed the highest recognition level in forest and unused land since 2015.

We provide the following selection suggestions for users. (1) Cross period observation and evaluation. Compared with other products, NLUD-C products provide different users with high-precision land cover data for China updated every five years with the high resolution, high recognition level, and high landform fitting degree. (2) Long time-series observation and evaluation. Both the CCI and MCD can provide historical images of land cover in China over the past two decades, but the overall accuracy of the MCD was slightly higher. (3) Different selection of land cover types. First, different products have different recognition levels for different land cover types. CCI should be used to analyze cropland and water, MCD to analyze grassland, and MCD and CGLS to analyze forest and unused land. Second, from a data classification system perspective, we suggest the use of data from an LCCS classification system to analyze cropland, water bodies, and construction land; data from an IGBP classification system should be used to analyze grassland and unused land.

## Data Availability

The datasets used and/or analysed during the current study available from the corresponding author on reasonable request.

## References

[1] Griggs DJ, Noguer M (2002). Climate change 2001: The scientific basis. Contribution of working group I to the third assessment report of the intergovernmental panel on climate change. Weather.

[2] Ran Y, Li X, Lu L (2010). Evaluation of four remote sensing based land cover products over China. Int. J. Remote Sens..

[3] Townshend J, Justice C, Li W, Gurney C, McManus J (1991). Global land cover classification by remote sensing: Present capabilities and future possibilities. Remote Sens. Environ..

[4] Yang LM, Zhu ZL (1999). The status quo and expectation of global and local land cover and land use RS research. J. Nat. Resourc..

[5] Xiao J, Shen Y, Ge J, Tateishi R, Tang C, Liang Y, Huang Z (2006). Evaluating urban expansion and land use change in Shijiazhuang, China, by using GIS and remote sensing. Landsc. Urban Plan..

[6] Loveland TR, Reed BC, Brown JF, Ohlen DO, Zhu Z, Yang L, Merchant JW (2000). Development of a global land cover characteristics database and IGBP DISCover from 1 km AVHRR data. Int. J. Remote Sens..

[7] Hansen MC, DeFries RS, Townshend JRG, Sohlberg R (2000). Global land cover classification at 1 km spatial resolution using a classification tree approach. Int. J. Remote Sens..

[8] Bartholome E, Belward AS (2005). GLC2000: A new approach to global land cover mapping from Earth observation data. Int. J. Remote Sens..

[9] Friedl MA, Sulla-Menashe D, Tan B, Schneider A, Ramankutty N, Sibley A, Huang X (2010). MODIS Collection 5 global land cover: Algorithm refinements and characterization of new datasets. Remote Sens. Environ..

[10] Defourny, P. *et al*. Land cover CCI: Product user guide version 2 (2016).

[11] Buchhorn, M. *et al*. Copernicus global land service: Land cover 100m: Version 3 Globe 2015-2019: Product user manual; Zenodo, Geneve, Switzerland (2020). 10.5281/zenodo.3938963.

[12] Jiyuan L, Mingliang L, Xiangzheng D, Dafang Z, Zengxiang Z, Di L (2002). The land use and land cover change database and its relative studies in China. J. Geogr. Sci..

[13] Herold M, Mayaux P, Woodcock CE, Baccini A, Schmullius C (2008). Some challenges in global land cover mapping: An assessment of agreement and accuracy in existing 1 km datasets. Remote Sens. Environ..

[14] Jung M, Henkel K, Herold M, Churkina G (2006). Exploiting synergies of global land cover products for carbon cycle modeling. Remote Sens. Environ..

[15] Tateishi R, Hoan NT, Kobayashi T, Alsaaideh B, Tana G, Phong DX (2014). Production of global land cover data—GLCNMO2008. J. Geogr. Geol..

[16] Latifovic R, Olthof I (2004). Accuracy assessment using sub-pixel fractional error matrices of global land cover products derived from satellite data. Remote Sens. Environ..

[17] McCallum I, Obersteiner M, Nilsson S, Shvidenko A (2006). A spatial comparison of four satellite derived 1 km global land cover datasets. Int. J. Appl. Earth Obs. Geoinf..

[18] Bai Y, Feng M, Jiang H, Wang J, Zhu Y, Liu Y (2014). Assessing consistency of five global land cover datasets in China. Remote Sens..

[19] Liu JY, Zhang ZX, Zhuang DF, Wang YM, Zhou WC, Zhang SW, Wu SX (2003). A study on the spatial-temporal dynamic changes of land-use and driving forces analyses of China in the 1990s. Geogr. Res..

[20] Yang Y, Xiao P, Feng X, Li H (2017). Accuracy assessment of seven global land cover datasets over China. ISPRS J. Photogramm. Remote. Sens..

[21] Bai Y, Feng M, Jiang H, Wang J, Liu Y (2015). Validation of land cover maps in China using a sampling-based labeling approach. Remote Sens..

[22] Luyuan J, Pengfeng X, Xuezhi F, Yun L, Liujun Z (2015). Assessment of Large-scale land cover datasets in Typical Areas of China based on sub-fractional error matrix. Remote Sens. Technol. Appl..

[23] Liu Q, Zhang Y, Liu L, Li L, Qi W (2019). The spatial local accuracy of land cover datasets over the Qiangtang Plateau, High Asia. J. Geog. Sci..

[24] Ren J, Yang J, Wu F, Sun W, Xiao X, Xia JC (2023). Regional thermal environment changes: Integration of satellite data and land use/land cover. iScience.

[25] Xie Q, Han Y, Zhang L, Han Z (2023). Dynamic evolution of land use/land cover and its socioeconomic driving forces in Wuhan, China. Int. J. Environ. Res. Public Health.

[26] Ma S, Wang LJ, Wang HY, Zhao YG, Jiang J (2023). Impacts of land use/land cover and soil property changes on soil erosion in the black soil region, China. J. Environ. Manage..

[27] Lu M, Wu WB, Zhang L, Liao A, Peng S, Tang H (2016). A comparative analysis of five global cropland datasets in China. Sci. China Earth Sci..

[28] Zhang D, Wang H, Wang X, Lü Z (2020). The reference data for accuracy assessment of the Global Forest Watch tree cover 2000 in China. Data Brief.

[29] Yang Z, Dong J, Liu J, Zhai J, Kuang W, ZhaoXiao GAX (2017). Accuracy assessment and inter-comparison of eight medium resolution forest products on the loess plateau, China. ISPRS Int. J. Geo-Inform..

[30] Lai L, Huang XJ, Yang H, Chuai X, Zhang M, Zhong T, Thompson JR (2016). Carbon emissions from land-use change and management in China between 1990 and 2010. Sci. Adv..

[31] Kang J, Wang Z, Sui L, Yang X, Ma Y, Wang J (2020). Consistency analysis of remote sensing land cover products in the tropical rainforest climate region: A case study of Indonesia. Remote Sens..

[32] Yang Y, Xiao P, Feng X, Li HX, Chang X, Feng W (2014). Comparison and assessment of large-scale land cover datasets in China and adjacent regions. J. Remote Sens..

[33] Liu C, Frazier P, Kumar L (2007). Comparative assessment of the measures of thematic classification accuracy. Remote Sens. Environ..

[34] Congalton RG, Gu J, Yadav K, Thenkabail P, Ozdogan M (2014). Global land cover mapping: A review and uncertainty analysis. Remote Sens..

[35] Gong P, Yu L, Li C, Wang J, Liang L, Li X, Zhu Z (2016). A new research paradigm for global land cover mapping. Ann. GIS.

[36] Hua T, Zhao W, Liu Y, Wang S, Yang S (2018). Spatial consistency assessments for global land-cover datasets: A comparison among GLC2000, CCI LC, MCD12, GLOBCOVER and GLCNMO. Remote Sens..

[37] Liang L, Liu Q, Liu G, Li H, Huang C (2019). Accuracy evaluation and consistency analysis of four global land cover products in the arctic region. Remote Sens..

[38] Zhao J, Dong Y, Zhang M, Huang L (2020). Comparison of identifying land cover tempo-spatial changes using GlobCover and MCD12Q1 global land cover products. Arab. J. Geosci..

[39] Belward AS, Valenzuela CR (1991). Spectral characteristics of vegetation, soil and water in the visible, near-infrared and middle-infrared wavelengths. Eurocours. Remote Sens..

